# Severe Cervical Myelopathy in a Patient With Intellectual Disability Successfully Managed With Combined Anterior and Posterior Fixation Surgery

**DOI:** 10.7759/cureus.66251

**Published:** 2024-08-06

**Authors:** Yoshinori Maki, Kenji Fukaya

**Affiliations:** 1 Neurosurgery, Hikone Chuo Hospital, Hikone, JPN; 2 Neurosurgery, Ayabe Renaiss Hospital, Ayabe, JPN

**Keywords:** cervical kyphosis, posterior cervical fixation, anterior cervical discectomy and fusion, cervical myelopathy, intellectual disability

## Abstract

Intellectual disability is a disorder characterized by lower developmental abilities in mental and physical performances. Due to advancements in healthcare management for patients with intellectual disabilities, the survival rate of these individuals has increased. Consequently, middle-aged patients with intellectual disabilities may present symptoms related to degenerative cervical spondylosis. However, there appear to be few reports focusing on this topic. A 52-year-old patient with intellectual disability was accompanied by his elderly parents to our hospital. The patient could not stand independently after experiencing motor weakness in the bilateral upper and lower extremities. Radiologically, cervical kyphosis and severe cervical cord compression were identified. After obtaining informed consent from the patient’s parents, cervical anterior and posterior fixation surgery was performed in two sessions to resolve cervical myelopathy. The patient was discharged from the hospital 45 days after the second operation. A year post-surgery, the patient could walk independently. With the long life expectancy of patients with intellectual disability, spinal degenerative diseases resulting in cervical myelopathy can significantly impact patients’ quality of life. Adequately examining, diagnosing, and surgically managing the patient can lead to improved status for patients with intellectual disability.

## Introduction

Intellectual disability, estimated to affect 1% of the population, manifests as a broad impairment of neurological functions encompassing intellectual and adaptive abilities [1,2]. Advanced healthcare management for individuals with intellectual disabilities has significantly improved their survival rates, preventing premature mortality that may have occurred before adolescence [1]. Despite these advancements, as individuals with intellectual disabilities age, their quality of life may be compromised by degenerative spinal conditions, akin to challenges faced by individuals without intellectual disabilities.

Cervical degenerative spinal lesions can result in cervical myelopathy. Cervical myelopathy can manifest various symptoms, including sensory and motor disturbance, and these symptoms can decline the activities of daily living of patients with cervical myelopathy [3,4]. Therefore, logically, long-term surviving patients with intellectual disability can be bothered by cervical myelopathy.

However, limited literature exists exploring the intersection of intellectual disability and coexisting cervical degenerative diseases [5]. Therefore, it seems that the benefit of undergoing cervical fixation/decompression surgery for patients with intellectual disability has not been stressed to date. In this context, focusing on the efficacy of appropriate surgical management, we present a case involving an adult with intellectual disability accompanied by cervical myelopathy which was successfully treated with cervical fixation surgery. We also highlight the potential impact of cervical lesions on the status of individuals with intellectual disabilities.

## Case presentation

**Figure 1:**
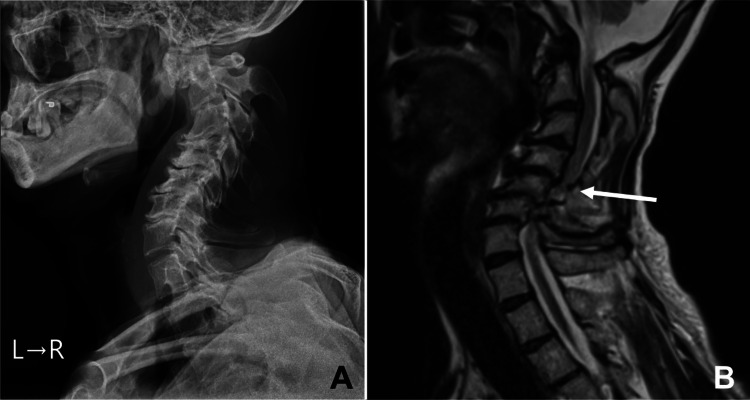
Preoperative radiological images. A lateral projection of the X-ray image shows cervical kyphosis (A). Severe cervical spinal cord compression can be observed (white arrow) (B).

A 52-year-old man presented to our emergency room, complaining of sudden motor weakness in the upper and lower extremities. He had a history of intellectual disability since childhood. Throughout his educational journey from primary to high school, he attended specialized classes tailored to individuals requiring additional support. Post-graduation, he resided in a group home, sustaining himself by collecting recyclable materials. Accompanied by his elderly parents, the patient exhibited an inability to stand due to sudden-onset motor weakness in both the upper (Manual Muscle Testing of 3) and lower extremities (Manual Muscle Testing of 2) that manifested 10 days prior. Additionally, he reported sensory disturbances and increased deep tendon reflexes. Radiological assessments to screen an intracranial or a spinal lesion unveiled findings of cervical kyphosis and severe cervical cord compression, indicative of potential cervical myelopathy (Figure 1).

**Figure 2:**
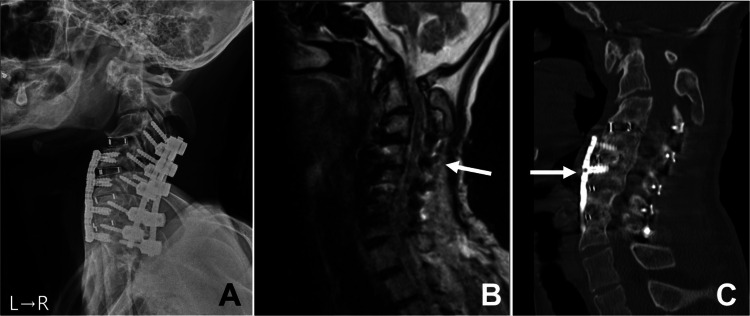
Postoperative radiological images. After anterior cervical discectomy and fusion from C3 to C7 and posterior fixation (A), the cervical spinal cord was decompressed (white arrow) (B). Three months after surgery, a bony fusion of the surgical regions was obtained (white arrow) (C).

Given the clinical presentation, surgical intervention was deemed necessary for decompression. Initially hesitant due to concerns over the surgical risks, the patient’s parents ultimately consented to the procedure upon understanding the potential consequences of inaction. As the neurological symptoms of the patient resulted in severe cervical kyphosis, an anterior and posterior combined approach seemed necessary. Thus, we planned an anterior and posterior combined approach in two sessions to resolve the cervical lesion. First, the patient underwent anterior cervical discectomy and fusion spanning C3 to C7. A plate was placed to reinforce the anterior fixation. Because of the anterior curvature of C3, stable and rigid placement of a plate covering C3 to C7 was difficult. Hence, the plate was finally placed from C4 to C7. This session was followed by posterior cervical decompression and fixation from C3 to T1 (Figure 2).

The postoperative recovery progressed smoothly, although thorough neurological evaluation utilizing standardized questionnaires was limited due to the patient’s limitations. After the second operation, the patient underwent rehabilitation therapy to improve his abilities of daily living. Postoperatively, any remarkable complications did not occur. Following rehabilitation therapy, the patient was discharged 45 days post-surgery and is currently undergoing follow-up care at an outpatient clinic, where he exhibits independent ambulation one-year post-surgery.

## Discussion

We described an adult case of intellectual disability accompanied by cervical myelopathy. The symptoms resulting from cervical myelopathy were successfully resolved with anterior and posterior cervical fixation surgery. A limited number of studies have described patients with intellectual disability and concomitant cervical myelopathy [5].

Previously, our coauthor reported a case of a patient with intellectual disability who underwent cervical laminoplasty for traumatic cervical spinal cord injury [5]. In the previous case, due to difficulties in neurologically interviewing and examining the patient, an initial diagnosis of sick sinus syndrome was made. However, cervical spinal cord injury was identified following bladder and rectal disturbances caused by motor weakness in the upper and lower extremities [5].

Individuals with intellectual disabilities have been prone to falls since childhood due to insufficient static and dynamic balance abilities [6-8]. Given his impaired static and dynamic balance abilities, as well as involuntary movements associated with intellectual disability, our patient likely experienced a significant spinal deformity, irrespective of his age. While the manifestation of severe cervical deformity is often observed in patients with athetoid-type cerebral palsy, where anterior and posterior cervical fixation surgery is a recommended course of action due to involuntary head and neck movements and severe cervical deformity [9], our patient did not have the same pathology. Nonetheless, the implementation of anterior and posterior cervical fixation surgery proved to be effective in resolving his cervical myelopathy.

Considering the scarcity of literature focusing on patients with intellectual disability and degenerative cervical diseases, the decline in neurological functions in certain cases may be perceived as a typical course for individuals with intellectual disabilities, potentially masking underlying spinal lesions. The repercussions of inappropriate management of undiagnosed diseases on individuals with intellectual disabilities are emphasized [10]. Furthermore, accurate neurological evaluations of individuals with intellectual disabilities can present challenges [11]. In our case, a neurological examination based on a questionnaire was unattainable.

The patient resided in a group home and received intermittent care from his elderly parents. However, with the eventual demise of his parents, the patient would lack their support. Thus, ensuring the maintenance of his quality of life and daily living abilities became essential. Despite initial parental reluctance toward surgical intervention, they eventually consented to improve the patient’s well-being. The successful implementation of anterior and posterior cervical fixations in two sessions led to the patient’s recovery, enabling him to independently ambulate a year post-surgery.

As demonstrated in our case, the comprehensive examination and surgical management of individuals with intellectual disabilities to rule out spinal lesions are imperative when neurological functions begin to decline. Surgery should be considered to sustain or enhance the patients’ daily living activities. We performed anterior cervical discectomy and fusion for four levels (C3-C7). Although this surgical procedure has the advantage of effectively improving preoperative neurological symptoms, perioperative complications such as dysphagia, hoarseness, hematoma, nerve palsy, and revision surgery were reported [12]. Therefore, the indication of surgical approaches should be carefully evaluated based on the patient’s condition and surgeon’s skill.

Because this is a single case report followed during a limited period, the long-term outcome of surgical treatment should be also evaluated. Besides, similar cases should be accumulated in further studies to standardize operative treatment for patients with coexistent intellectual disability and cervical lesions.

## Conclusions

Our case highlights the successful management of cervical myelopathy in an adult with an intellectual disability through anterior and posterior fixation surgery. It is imperative to recognize the potential existence of undiagnosed cervical degenerative diseases in individuals with intellectual disabilities in clinical practice. Timely assessment, diagnosis, and consideration of surgical interventions tailored to individual circumstances are paramount to optimizing patient outcomes and ensuring holistic care.
